# Self-Reported Oil Spill Exposure and Pregnancy Complications: The GROWH Study

**DOI:** 10.3390/ijerph14070692

**Published:** 2017-06-27

**Authors:** Emily W. Harville, Arti Shankar, Leah Zilversmit, Pierre Buekens

**Affiliations:** 1 Department of Epidemiology, Tulane School of Public Health and Tropical Medicine, 1440 Canal St. Ste. 2000 #8318, New Orleans, LA 70112-2715, USA; lzilvers@tulane.edu (L.Z.); pbuekens@tulane.edu (P.B.); 2 Department of Global Biostatistics and Data Science, Tulane School of Public Health and Tropical Medicine, New Orleans, LA 70112-2715, USA; sarti@tulane.edu

**Keywords:** oil spill, diabetes, gestational, hypertension, pregnancy-induced, nausea

## Abstract

Adverse infant outcomes often rise in the aftermath of disaster, but few studies have assessed the effects of disaster on maternal health. 1091 southern Louisiana women were interviewed about their pregnancy history, including pregnancy complications. Associations between oil spill exposures and gestational diabetes, hypertensive disorders, and nausea/vomiting were assessed for all reported pregnancies. 631 women had a pregnancy both before and after the oil spill. Generalized estimating equations (logistic regression) with adjustment for confounders were used. To assess possible unmeasured confounding, instead of considering oil spill exposure as a time-varying exposure, women were defined as oil spill-exposed or not. If oil spill-exposed women were equally prone to complications in pregnancies that occurred prior to the oil spill as after it, it was considered that any associations were likely due to selection or reporting issues. Women who reported oil spill exposure, particularly loss of use of the coast, were more likely to report gestational diabetes; however, the level of association was similar for pregnancies before and after the spill (*p* for interaction >0.10 and odds ratios (ORs) for pregnancies prior to the spill > than those after the spill). No associations were found between oil spill exposure and hypertensive disorders. This analysis does not suggest an increased risk of pregnancy complications associated with exposure to the oil spill; however, future studies should assess exposure and outcomes prospectively and clinically instead of relying on self-report.

## 1. Introduction

Pregnant women are often considered a vulnerable population during disasters, both natural and chemical [1], and for this reason, the effects of the Gulf oil spill on pregnant women are of particular concern. Although a large number of studies have examined the effect of disaster on birth outcomes, such as low birthweight or preterm birth [2], and an additional line of research examines its effects on the infants [3], relatively few studies have assessed how disaster might affect maternal health. In terms of major medical complications, gestational diabetes (GDM) and hypertensive disorders of pregnancy (including pre-eclampsia (PE) and pregnancy-induced hypertension (PIH)) have been examined in a few studies, with mixed results [4,5,6,7,8]; some studies show an increase, others show no effect.

Any disaster could be hypothesized to trigger such complications via endocrine or behavioral pathways: stress is associated with increased blood pressure and glucose levels [9,10,11]. Disaster may also change the availability of healthy foods and motivation available for healthy eating patterns, or cause shifts in smoking prevalence [12,13,14,15,16,17,18,19]. When considering effects of an oil spill specifically, chemical exposures are also of concern [20]. Again, evidence is limited, but some pollutants associated with the oil spill, including heavy metals and products of combustion, have been associated with GDM and PIH [21,22,23]. Although few studies have considered nausea/vomiting for pregnancy specifically, studies of workers in the petrochemical industry [24] and oil spill cleanup workers have reported nausea/vomiting (or dizziness) [25].

In this analysis, we examine the association between self-reported exposure to the physical and social/economic effects of the Gulf oil spill, and pregnancy complications.

## 2. Methods

### 2.1. Participants

The Gulf oil spill occurred between April and September 2010. It was the largest accidental oil spill in history, and had a series of effects starting with the death of 11 rig workers; release of 3.19 million barrels into the Gulf of Mexico; contamination of the water, coasts, and estuaries on the Gulf Coast; death of wildlife; and fishing ground closures and an offshore drilling moratorium that resulted in loss of livelihood [26]. The GROWH (Gulf Resilience on Women’s Health) Study recruited from 2011 to 2016. Women were recruited from prenatal, health, and Women, Infants, and Children (WIC) clinics; day care centers; and community events and gathering places in southeastern Louisiana (targeting West Bank of Jefferson and Orleans Parishes, and Lafourche, Plaquemines, St. Bernard, and Terrebonne Parishes). Eligibility criteria include: aged 18–45, living in the Gulf area during the oil spill, and, if pregnant, carrying a singleton gestation. Women were interviewed, completed a questionnaire (usually at the same visit, although taking it home and returning it by mail was allowed), and provided saliva and blood samples. 1650 filled out at least one questionnaire or interview, including 460 women who were pregnant at the time of the interview. 1091 women had data on at least one oil spill exposure and pregnancy complications on at least one post-oil spill pregnancy. Included women were more likely to be older, have a higher Body Mass Index (BMI), pregnant, white or black, and smokers (*p* < 0.05); no differences were seen in parish of residence or marital status. 631 had data on complications in a pregnancy both before and after the spill.

### 2.2. Measures

#### 2.2.1. Exposures

Oil spill experience was measured with questions from several sources, including questions about: (1) a participant’s involvement in work on the clean-up and contact with oil, taken from the Gulf Workers’ Study [27]; (2) direct exposure to the oil spill, taken from studies performed after the Exxon Valdez spill [28]; and (3) the social and economic effects of the oil spill, from a previous study (GUMBO, R03 NR012052). (Specific questions are provided in the Supplementary Materials.) Confirmatory factor analysis was used to see if the patterns of grouping of similar response questions matched the underlying latent constructs: financial/income consequences; direct contact with oil (both dichotomized as any/none and none/some/a lot); oil spill-related trauma (damage to people or own property); loss of use of the coast (damage to areas where one or one’s family fishes, boats, or goes to the coast or beach); and involvement in litigation. In addition, separate variables for any exposure to the oil spill (0 versus 1) and total exposure to the oil spill (sum of the above individual experiences: money, direct contact, trauma, loss of use, and litigation, weighted equally; theoretical range was 0 to 10; range in this sample was 0 to 9).

#### 2.2.2. Outcomes

Each woman was also asked for a reproductive history including up to 8 pregnancies. Questions for participants included date of/age at each pregnancy and complications of the pregnancy: “During (this/that) pregnancy, did you ever develop (check all that apply): hypertension or high blood pressure, pre-eclampsia or toxemia, diabetes or high blood sugar” and “During (this/that) pregnancy, did you have frequent nausea/vomiting?”. Hypertensive disorders were defined as a “yes” response to either “pre-eclampsia or toxemia” or “hypertension or high blood pressure”. These questions were asked regardless of the outcome of the pregnancy (livebirth/miscarriage/stillbirth/etc.); the analysis for gestational diabetes and hypertensive disorders was limited to pregnancies carried to term (miscarriages, induced abortions, and those still pregnant omitted). The analysis of nausea/vomiting included all pregnancies, as this symptom occurs most commonly early in pregnancy.

The date of each pregnancy (based on estimated last menstrual period) was determined to occur before (prior to 20 April 2010), or during or after the oil spill (on or after 20 April 2010). If the precise date of the start or end of pregnancy was not known and it was estimated to have occurred within 6 months of the oil spill, it was omitted from the analysis.

Statistical analysis. First, all pregnancies for a given woman were examined. Pregnancies prior to the oil spill were categorized as unexposed, while pregnancies after the oil spill categorized as exposed or unexposed, depending on the particular indicator under study. Generalized estimating equations (GEE) were used to control for correlation within woman (up to 8 pregnancies/woman). Second, a similar GEE model was run, limited to women who had both a pre- and post-oil spill pregnancy. Both unadjusted and adjusted logistic regression were used, with adjustment for age at pregnancy (continuous), race (black/not black), gravidity at that pregnancy, BMI (continuous), income (ordinal), education (ordinal), smoking (dichotomous), and weight gain during pregnancy (continuous) (details of categories in Table 1). Multiple imputation was used to account for missing covariate data. Finally, a model was run categorizing women based on their reported exposure to the oil spill, with an interaction between pregnancies occurring prior to and those occurring after the oil spill. If exposure was equally or more predictive of outcomes prior to the oil spill (i.e., the interaction was not significant), it was considered that any associations were likely due to unmeasured confounding. Thus, the pregnancies before the oil spill serve as negative controls [29].

Sensitivity analyses limited the analysis to those whose pregnancies occurred within two years of the oil spill (*n* = 695 overall and 573 with pregnancies both before and after).

In addition, when possible, pregnant women had their medical records abstracted. Records were located for 386 pregnant women, and recorded instances of gestational diabetes or hypertensive disorders were abstracted. Two analyses were run: one, including only records that specified clearly positive or negative results for these disorders, and two, assuming that any records not marked as having gestational diabetes or a hypertensive disorder were negative for those disorders. This sample size was too small for a pre-post or limited-time sensitivity analysis.

The study methods were approved by the Institutional Review Boards of Tulane University, Ochsner, and WIC, and all participants provided written informed consent (Tulane IRB # 239911).

## 3. Results

The study population was predominantly <30 years old, black, and with high BMI (Table 1). Approximately half reported some sort of exposure to or effect of the oil spill, most often financial. GDM and PE were reported by 7–9%; hypertension by 21%, and nausea/vomiting by 70–75%.

Any exposure to the oil spill was associated with increased risk of GDM (aOR 1.84, 95% CI: 1.14–2.99; Table 2), but no individual contributor could be identified as contributing strongly to that. No associations were found with hypertensive disorders (Table 3). Overall, increased nausea/vomiting post-oil spill was found (aOR 1.60, 95% CI: 1.12–2.27; Table 4) in the sample with both pre- and post-oil spill pregnancies, and particularly with income loss (aOR 1.75, 95% CI: 1.07–2.85).

Overall, few interactions were found between timing of pregnancy and oil spill exposure, suggesting that reported oil spill exposure did not more strongly predict these outcomes for pregnancies that occurred after the oil spill. In the cases where there was a suggestion of an interaction (*p* < 0.15), effects were stronger for pregnancies prior to the oil spill for gestational diabetes (Table S1). The relationship between contact with the oil and nausea/vomiting was statistically different, and stronger, for pregnancies after the spill, but was not statistically significant overall (pre-oil spill aOR 0.83, 95% CI: 0.53–1.29; post-oil spill, aOR 1.48, 95% CI: 0.81–2.69; *p* for interaction = 0.05; Table S2).

The medical records analysis did not indicate any associations with gestational diabetes (Table S3). Hypertensive disorders were more common only in those reporting contact with oil (aOR 3.13, 95% CI: 1.05–9.35).

## 4. Discussion

This analysis did not find evidence of strong associations between oil spill exposure and pregnancy complications, with what few associations there were largely being equally strong for pregnancies occurring prior to the oil spill as after. Few studies have previously assessed the association of maternal pregnancy complications with either chemical or natural disaster, and results are conflicting. While exposure to the 2010 Chile earthquake was associated with increased gestational diabetes if exposed in the first semester (OR 3.9, 95% CI: 1.0–15.5) [4], the exposure to the 2003 Canberra Wildfires were not [4]. An increase in eclampsia was noted after the 1997 Red River flood in North Dakota [7], but a decline in eclampsia was noted after Hurricane Katrina [8]. Individual differences may contribute to the risk: stress and certain coping styles in the aftermath of Hurricane Katrina were associated with gestational diabetes and pregnancy-induced hypertension [6]. We are not aware of any studies specifically addressing nausea/vomiting during pregnancy; a study of pregnant women near the Hebei Spirit oil spill in South Korea found an increase in minor somatic symptoms, although not nausea/vomiting [30]. Nausea/vomiting during pregnancy is associated with stress and psychological symptoms, although the direction of the association is not clear [31,32,33]. Our results are not inconsistent with an increased risk, but do not demonstrate that definitively.

Biological and behavioral pathways for an effect on pregnancy complications can be hypothesized. Disaster has been associated with changes in glucose levels, both in diagnosed diabetics [34,35,36] and undiagnosed or unaffected people [10]. Work in the petrochemical industry has also been associated with higher blood glucose [37]. Some studies have also found increased blood pressure with exposure to disaster stress or exposure to petroleum or related products [38,39,40,41]. We did find an increase in recorded hypertensive disorders in the medical records of the pregnant women among those who reported contact with oil, although not with other oil spill exposures. This association should be investigated in more detail in studies with more detailed exposure and outcome measures. A less regulated diet, less physical activity, and weight gain—strong risk factors for GDM and PIH—are plausible consequences of social and economic stress. A lack of effect is also plausible. Women in this study were interviewed largely two to four years after the oil spill itself, so short-term symptoms such as nausea may not have been accurately remembered. Nausea is quite common during pregnancy in any case; the oil spill may not have had a strong enough effect to distinguish it from the background levels, and we did not ask about severity. Most women did not live near enough to the oil spill to smell it, which has been associated with reporting physical symptoms [42]. Truly assessing whether an oil spill was exacerbating mild cases of nausea would probably require detailed, prospective data collection.

Strengths of the study include a relatively unselected population, with no particular reason to over or under-report exposures and outcomes; a reasonably large sample size; and the opportunity for examining pregnancies that occurred prior to the oil spill as a negative control. We performed as systematic and thorough an assessment of outcomes and exposures as possible within the study design; still, measurement error is likely present. Pregnancy complications are difficult to measure. Previous studies often use vital records data, which usually under-reports complications and has limited information on some important complications, like pre-eclampsia (only eclampsia—quite rare—is reported on the standard birth certificate. Pre-eclampsia is combined with pregnancy-induced hypertension [43]). Complications in this analysis were measured primarily via self-report. Previous studies have found maternal self-report to be accurate for reports of gestational diabetes (GDM) (specificity = 98%, sensitivity = 92%) [44] and highly specific (>90%) for hypertensive disorders [45]. Nonetheless, self-report is unlikely to overlap completely with medical diagnosis. Besides simply mistaken or misunderstood recall of complications, women who had hypertension prior to the pregnancy, or who were told that their blood pressure seemed a little high at a prenatal visit, or who were asked to come back after glucose tolerance screening but were not diagnosed with diabetes, might have correctly answered yes to the questions as posed. In most cases, women were interviewed during pregnancy prior to likely onset of GDM or hypertensive complications (thus, those pregnancies are omitted from those analyses). Nausea and vomiting, unless severe enough to require hospitalization, have no other plausible source of information beyond self-report. In certain types of disaster, disruption of the health care system or over-diagnosis could be causes for bias; these are unlikely for this particular topic. Still, recall bias, or reporting error, is possible. If not connected with exposure, the most likely consequence of this error is bias towards the null.

Other limitations of the study include the fact that the post-oil spill and pre- and post-samples are different, most obviously by conditioning on gravidity, and the age and gravidity at a second pregnancy is by definition larger than at the first. Thus, the earlier pregnancies are an imperfect control and residual confounding is possible, although previous analysis of ours found no association between oil spill exposure and fertility [46]. Similarly, oil spill exposure is self-reported, which likely leads to misclassification or, at least, inexactness in measurement; the most likely consequence of this is bias towards the null. Too few women were pregnant during the spill to examine timing of exposure relative to the pregnancies. Likewise, many analyses were conducted in the course of this study, and chance findings are a possibility. Similarly, the sample size was insufficient to detect weaker associations.

## 5. Conclusions

This study highlights the need for infrastructure for rapid data collection in the aftermath of disaster [47]. Especially for an exposure with no clear biomarker, such as an oil spill, exposure assessment is limited to self-report or ecologic measures. Similarly, health outcomes with no well-defined registry or for which self-report is limited require immediate, prospective data collection to be accurately assessed. The lack of conclusiveness of our results supports the need for data collection efforts that allow for decisively null or positive conclusions. Future studies should attempt to establish data collection systems to collect prospective data as soon as possible, including systems that take advantage of existing medical records and other routine data collection.

## Figures and Tables

**Table 1:** Participants in the GROWH study of oil spill, 2011–2016.

	Participants with Post-Oil Spill Pregnancy (*n* = 1091)		Participants with Pre- and Post-Oil Spill Pregnancy (*n* = 631)	
Participant characteristic	***N***	**%**	***N***	**%**
age at interview				
18–25	387	36.9	102	16.8
>25–30	329	31.3	223	36.8
>30–35	211	20.1	173	28.6
>35	123	11.7	108	17.8
race				
white	314	29.5	178	29.0
black	652	61.3	381	62.1
other	98	9.2	55	9.0
pre-pregnancy or current BMI				
≥20	70	6.7	38	6.3
>20–25	242	23.2	121	20.0
>25–30	270	25.8	158	26.2
>30	463	44.3	287	47.5
education				
High school or less	560	52.6	328	53.3
Some college/associates	423	39.8	250	40.6
College or more	81	7.6	38	6.2
married or living with partner				
yes	438	40.9	265	42.7
no	632	59.1	355	57.3
income category				
≤$20 K	513	49.1	296	48.1
>$20 K–40 K	344	32.9	206	33.5
>$40	188	18.0	113	18.4
pregnant at time of interview				
yes	389	35.7	201	31.9
no	702	64.3	430	68.2
parity				
0	103	9.7	7	1.1
1	334	31.6	91	14.6
2+	621	58.7	524	84.2
coastal zip code				
yes	205	19.8	125	20.6
no	832	80.2	483	79.4
Smoke in last 2 years				
yes	312	28.8	190	30.3
no	770	71.2	437	69.7
any oil spill exposure	533	49.0	325	51.7
high income loss	276	25.3	167	26.5
contact with oil	185	17.0	111	17.7
trauma/property	62	5.8	44	7.1
loss of use of coast	393	37.2	234	38.2
involvement in litigation	256	23.5	154	24.4
GDM at post-OS preg	75	7.8	50	9.2
PE at post-OS preg	80	8.3	42	7.8
PIH at post-OS preg	196	21.6	108	21.1
Nausea/vomiting at post-OS preg	624	70.3	368	72.6

GDM, gestational diabetes mellitus; OS, oil spill; PE, pre-eclampsia; PIH, pregnancy-induced hypertension.

**Table 2:** Oil spill exposure and gestational diabetes.

Indicator of Oil Spill Exposure	Participants with Any Post Oil-Spill Pregnancy (*n* = 962, 2059 Pregnancies)				Participants with Both A Pre- and Post-Oil Spill Pregnancy (*n* = 616, 1503 Pregnancies) **			
	Unadjusted		Adjusted *		Unadjusted		Adjusted *	
	OR	95% CI	OR	95% CI	OR	95% CI	OR	95% CI
post oil spill	1.75	1.24–2.49	1.20	0.77–1.85	1.39	1.16–1.66	0.80	0.48–1.35
any exposure	1.93	1.25–2.99	1.84	1.14–2.99	2.15	1.26–3.66	1.86	1.01–3.41
income loss	1.40	0.85–2.33	1.11	0.65–1.91	1.97	1.11–3.51	1.40	0.74–2.62
contact with oil	1.52	0.87–2.66	1.09	0.55–2.18	1.39	0.69–2.77	0.57	0.23–1.40
trauma	1.56	0.64–3.78	0.88	0.27–2.91	1.27	0.43–3.77	0.69	0.19–2.48
coast	1.63	1.06–2.50	1.29	0.80–2.09	1.98	1.21–3.27	1.23	0.69–2.21
litigation	1.00	0.58–1.74	0.82	0.44–1.50	1.21	0.65–2.27	0.79	0.38–1.64
total oil spill exposure								
low	1.00		1.00		1.00		1.00	
medium-low	1.52	0.94–2.47	1.34	0.80–2.24	1.56	0.85–2.89	1.05	0.55–1.97
medium-high	1.54	0.80–2.97	1.15	0.53–2.50	2.10	0.98–4.47	1.26	0.52–3.07
high	1.75	0.85–3.62	1.18	0.50–2.81	1.96	0.92–4.19	1.02	0.39–2.64

* adjusted for age, race, gravidity, BMI, income, education, smoking, year of interview, weight gain during pregnancy; ** these analyses are on a subset of the women included in the first columns.

**Table 3:** Oil spill exposure and hypertensive disorder.

Indicator of Oil Spill Exposure	Participants with Any Post Oil-Spill Pregnancy (*n* = 962, 2022 Pregnancies)				Participants with Both A Pre- and Post-Oil Spill Pregnancy (*n* = 628, 1873 Pregnancies)			
	Unadjusted		Adjusted *		Unadjusted		Adjusted *	
	OR	95% CI	OR	95% CI	OR	95% CI	OR	95% CI
post oil spill	1.14	0.94–1.37	1.03	0.79–1.34	1.05	0.95–1.16	0.75	0.53–1.08
any exposure	1.11	0.85–1.45	1.14	0.85–1.52	1.10	0.80–1.52	1.18	0.81–1.70
income loss	1.24	0.93–1.65	1.23	0.88–1.73	1.38	0.98–1.96	1.34	0.84–2.15
contact with oil	1.21	0.86–1.70	1.06	0.69–1.61	1.43	0.96–2.14	1.02	0.57–1.83
trauma	0.77	0.44–1.35	0.73	0.38–1.40	0.94	0.51–1.73	0.98	0.42–2.26
coast	1.17	0.91–1.51	1.11	0.81–1.51	1.17	0.85–1.59	0.97	0.63–1.49
litigation	1.04	0.75–1.43	0.87	0.60–1.25	1.22	0.82–1.80	1.08	0.66–1.77
total oil spill exposure								
low	1.00		1.00		1.00		1.00	
medium-low	1.52	0.94–2.47	0.83	0.58–1.18	0.90	0.61–1.33	0.71	0.43–1.16
medium-high	1.54	0.80–2.97	1.05	0.67–1.65	1.15	0.72–1.85	0.89	0.47–1.70
high	1.75	0.85–3.62	1.25	0.71–2.22	1.74	1.05–2.90	1.39	0.67–2.90

* adjusted for age, race, gravidity, BMI, income, education, smoking, year of interview, weight gain during pregnancy.

**Table 4:** Oil spill exposure and nausea/vomiting.

Indicator of Oil Spill Exposure	Participants with Any Post Oil-Spill Pregnancy (*n* = 1084, 2434 Pregnancies)				Participants with Both a Pre- and Post-Oil Spill Pregnancy (*n* = 628, 1826 Pregnancies)			
	Unadjusted		Adjusted *		Unadjusted		adjuSted *	
	OR	95% CI	OR	95% CI	OR	95% CI	OR	95% CI
post oil spill	1.30	1.11–1.52	1.23	0.98–1.54	1.17	1.70–1.27	1.60	1.12–2.27
any exposure	1.09	0.87–1.37	1.12	0.88–1.44	1.14	0.86–1.52	1.33	0.96–1.85
income loss	1.36	1.06–1.74	1.15	0.86–1.54	1.46	1.08–1.97	1.75	1.07–2.85
contact with oil	1.37	1.01–0.85	1.26	0.90–1.77	1.46	1.02–2.09	1.70	0.98–2.95
trauma	0.96	0.59–1.56	1.05	0.59–1.87	1.03	0.50–1.81	1.14	0.53–2.46
coast	1.23	0.99–1.53	1.13	0.88–1.46	1.26	0.97–1.63	1.40	0.93–2.10
litigation	0.97	0.75–1.25	0.82	0.61–1.11	1.10	0.81–1.49	1.06	0.68–1.65
total oil spill exposure								
low	1.00		1.00		1.00		1.00	
medium-low	1.06	0.83–1.33	0.99	0.75–1.31	1.18	0.89–1.57	1.11	0.74–1.69
medium-high	1.38	1.00–1.90	1.22	0.84–1.76	1.31	0.88–1.95	1.58	0.82–3.05
high	1.11	0.72–1.70	0.94	0.57–1.53	1.23	0.75–2.00	1.35	0.66–2.74

* adjusted for age, race, gravidity, BMI, income, education, smoking, year of interview, weight gain during pregnancy.

## Supplementary Materials

The following are available online at www.mdpi.com/1660-4601/14/7/692/s1, Table S1: Oil Spill Exposure and Interaction with Gestational Diabetes among Women with Both a Pre- and Post-Oil Spill Pregnancy (616 Women, 1601 Pregnancies), Table S2: Oil Spill Exposure and Interaction with Nausea/Vomiting among Women with Both a Pre- and Post-Oil Spill Pregnancy (628 Women, 1820 Pregnancies), Table S3: Oil Spill Exposure and Complications Recorded in Medical Records, Oil Spill Experience questionnaire.
