# Supplementary material for: Self-Reported Oil Spill Exposure and Pregnancy Complications: The GROWH Study
Source: Int J Environ Res Public Health. 2017 Jun 27;14(7):692. doi: 10.3390/ijerph14070692 (PMC5551130; doi:10.3390/ijerph14070692)
Supplement: Supplementary file 1 [file ijerph-14-00692-s001.pdf]

Table S1. Oil Spill Exposure and Interaction with Gestational Diabetes among Women with Both a Pre- and Post-Oil Spill Pregnancy (616 Women, 1601 Pregnancies)									
Indicator of oil spill exposure	unadjusted				adjusted				
	pre		post		pre		post		*P* for interaction
	OR	95% CI	OR	95% CI	OR	95% CI	OR	95% CI	
any exposure	3.04	1.44–6.41	1.74	0.96–3.16	2.57	1.15–5.72	1.40	0.69–2.81	0.21
income	3.01	1.57–5.76	1.67	0.91–3.09	2.73	1.32–5.66	1.55	0.75–3.17	0.22
contact with oil	1.54	0.73–3.23	0.97	0.46–2.05	1.31	0.60–2.89	0.60	0.23–1.53	0.12
trauma	2.47	0.99–6.12	1.00	0.33–3.05	2.39	0.87–6.55	0.61	0.15–2.44	0.12
coast	3.23	1.62–6.45	1.63	0.91–2.91	2.71	1.28–5.72	1.41	0.71–2.83	0.12
litigation	1.24	0.60–2.57	0.77	0.38–1.58	1.23	0.56–2.68	0.78	0.36–1.69	0.43
total oil spill exposure									
low	1.00		1.00		1.00		1.00		
medium-low	3.38	1.44–7.93	1.21	0.60–2.43	3.03	1.28–7.18	1.17	0.54–2.56	
medium-high	1.48	0.47–2.49	1.54	0.66–3.61	2.24	0.78–6.40	1.54	0.59–4.04	
high	4.40	1.61–12.04	1.62	0.66–3.94	3.57	1.18–10.79	1.10	0.37–3.20	

Table S2. Oil Spill Exposure and Interaction with Nausea/Vomiting among Women with Both a Pre- and Post-Oil Spill Pregnancy (628 Women, 1820 Pregnancies)

Indicators of oil spill exposure	unadjusted				adjusted				p for interaction
	pre		post		pre		post		
	OR	95% CI	OR	95% CI	OR	95% CI	OR	95% CI	
any exposure	1.16	0.84–1.61	1.14	0.81–1.61	1.27	0.88–1.84	1.46	0.94–2.27	0.64
income	1.00	0.69–1.43	1.17	0.79–1.74	1.18	0.77–1.79	1.50	0.88–2.54	0.36
contact with oil	0.90	0.60–1.34	1.11	0.72–1.73	0.83	0.53–1.29	1.48	0.81–2.69	0.05
trauma	1.30	0.64–2.65	0.91	0.46–1.80	1.09	0.50–2.37	0.99	0.44–2.24	0.80
coast	1.00	0.72–1.40	0.97	0.68–1.39	1.11	0.76–1.61	1.14	0.72–1.80	0.86
litigation	0.96	0.66–1.39	0.81	0.55–1.19	1.03	0.67–1.57	0.83	0.51–1.36	0.51
total oil spill exposure									
low	1.00		1.00		1.00		1.00		
medium-low	1.01	0.69–1.47	0.85	0.57–1.27	1.25	0.83–1.89	0.83	0.51–1.35	
medium-high	1.03	0.64–1.66	0.95	0.57–1.60	1.06	0.61–1.84	1.23	0.60–2.51	
high	0.86	0.49–1.52	0.82	0.45–1.51	0.95	0.49–1.84	1.02	0.46–2.24	

Table S3. Oil Spill Exposure and Complications Recorded in Medical Records								
	gestational diabetes				hypertensive disorders			
	unadjusted		adjusted [b]		unadjusted		adjusted *	
Indicator of oil spill exposure	OR [a]	95% CI	OR	95% CI	OR	95% CI	OR	95% CI
any exposure	1.09	0.43–2.79	1.26	0.42–3.83	1.63	0.68–3.92	2.03	0.73–5.62
	1.11	0.44–2.82	1.28	0.43–3.81	1.60	0.67–3.82	1.89	0.70–5.07
income loss	1.57	0.59–4.18	1.79	0.60–5.37	1.20	0.47–3.07	1.30	0.46–3.65
	1.54	0.58–4.05	1.73	0.59–5.10	1.13	0.45–2.86	1.18	0.43–3.22
contact with oil	0.62	0.14–2.78	0.22	0.02–1.93	3.10	0.94–10.24	3.13	1.05–9.35
	0.65	0.14–2.90	0.21	0.02–1.84	2.73	1.05–7.07	3.07	1.06–8.90
coast	1.64	0.61–4.41	1.76	0.58–5.41	2.49	0.95–6.52	1.38	0.52–3.68
	1.70	0.64–4.54	1.85	0.61–5.57	1.14	0.48–2.69	1.30	0.50–3.38
litigation	1.09	0.37–3.15	1.03	0.33–3.25	0.86	0.30–2.42	0.83	0.27–2.51
	1.05	0.37–3.02	1.00	0.33–3.08	0.80	0.29–2.24	0.75	0.26–2.20

[a] first estimate includes only those with complete records on the complication of interest. Second estimate includes assumes all those without a complication explicitly marked are negative for the complication; [b] adjusted for age, gravidity, BMI, income, education, smoking, year of interview, weight gain during pregnancy.

Oil Spill Experience

Please indicate your experience regarding the Deepwater Horizon Oil Spill, which occurred in the Gulf of Mexico beginning from April 20th 2010. Consider your experiences during the initial event, the continuing spill, and the time until the present.

Direct environmental exposure	
Did you do any work around the time of the oil spill cleanup that was needed because of the spill? Remember that this work could include jobs like cooks or food service personnel; fork lift drivers; security personnel; health & safety personnel; foremen; drivers; and so on.	Yes/No
Did/do you spend any time for any reason….on the beaches?	Yes/No
…in an area where workers were/are deconning vessels or equipment?	Yes/No
…in any other place where oil, oily materials, or chemicals were/are being used?	Yes/No
Did you handle any wildlife, whether alive or dead, during the cleanup?	Yes/No
I came in contact with oil and other debris during cleanup activities of the oil spill	Yes/No
I came into contact with oil during activities such as fishing, hunting, or gathering activities.	Yes/No
A member of my household [*if necessary*: those living in the same house as me] came into contact with oil during oil spill cleanup or activities such as hunting, fishing or gathering.	Yes/No

I came into contact with oil during the following time period (check all that apply):

April 2010
May 2010
June 2010
July 2010
Sept 2010
Oct 2010
Nov 2010
Dec 2010
Jan 2010
Feb 2010
Mar 2010
April 2010
After April 2010

Economic and social consequences

Before the spill, did you or anyone in your household use areas along the coast that were affected by the spill?	Yes/No
Did you have any property that was lost or damaged because of the oil spill or cleanup?	Yes/No
Did the oil spill cause any damage to the areas you or members of your household fish commercially?	Yes/No
Has the oil spill directly affected the hunting, fishing, or gathering activities of any members of this household?	Yes/No
Was someone close to you injured or killed in the Deepwater Horizon explosion, the oil spill, or the cleanup?	Yes/No
Have you or someone close to you lost income or earning capacity due to the oil spill? For example, did you lose your job or have your hours cut because of the spill? This might apply to fishermen, workers in seafood industry, workers in hotels or restaurants, or others. In terms of income, did you lose:	Nothing/a little/some/a lot
I have been contacted by attorneys.	Yes/No
I believe that I need legal representation to deal with the oil spill.	Strongly agree - strongly disagree
There have been a lot of demands made by litigation.	Strongly agree - strongly disagree
I have had unpleasant experiences or memories of the litigation.	Strongly agree- Strongly disagree
